# A Case Series on the Spectrum of Complications Observed in Kyasanur Forest Disease

**DOI:** 10.7759/cureus.59971

**Published:** 2024-05-09

**Authors:** Vineeth Gladson, Sheela Mathew, Benny J., Adarsh Aayilliath K., Jayesh Kumar P., Rajasi R.S.

**Affiliations:** 1 Internal Medicine, Government Medical College Kozhikode, Kozhikode, IND; 2 Infectious Diseases, Kunhitharuvai Memorial Charitable Trust Medical College, Kozhikode, IND; 3 Infectious Diseases, Amala Institute of Medical Sciences, Thrissur, IND; 4 Community Medicine, Government Medical College Kozhikode, Kozhikode, IND

**Keywords:** myocarditis, hlh, monkey fever, neurological complications, kfd, kyasanur forest disease

## Abstract

Background

Kyasanur Forest Disease (KFD) has emerged as an important differential diagnosis of febrile illness for physicians caring for patients in the Western Ghats of South India over the last decade.

Aim

This study seeks to familiarize physicians with the clinical presentation and the clinical, laboratory and imaging findings of the various complications of KFD. It also seeks to review the literature on the complications of KFD described.

Material and methods

This was a records-based retrospective study of the patients with KFD referred for tertiary care management to Government Medical College Kozhikode, Kerala over 11 years, from January 2013 to December 2023.

Results

A total of 12 case records were obtained and analysed. All the patients in this case series belonged to tribal ethnic groups enhancing its social significance. The complications of KFD (as calculated in the 11 patients for whom all the records were available) were altered sensorium (nine, 82%), persistent shock (seven, 64%), Acute Respiratory Distress Syndrome (ARDS)/pneumonitis (six, 55%), encephalitis (six, 55%), myocarditis (six, 55%), bleeding manifestations (six, 55%), hepatitis (six, 55%), acute kidney injury (four, 36%), rhabdomyolysis (three. 27%), hemophagocytic lymphohistiocytosis (HLH) (two, 18%), stress hyperglycaemia (two, 18%), pancreatitis (one, 9%), peritonitis (one, 9%). The case fatality rate in this series was 42%( n=5/12). An autopsy was done on one patient which showed congested and oedematous lungs with subpleural haemorrhage. Petechial haemorrhages were noted in the liver, spleen and kidney. The total leucocyte count was lower than 2500 c/mm3 in 10 (90%) patients. Out of the four patients in whom serum ferritin was tested, it was elevated (above 500 ng/ml) in all patients; and was above 1000 ng/ml in three patients. Hemophagocytic lymphohistiocytosis was diagnosed in two patients. This is a unique finding of our series. Both of these patients succumbed to the illness. A cerebrospinal fluid study was done in six patients and revealed normal values except in one patient. Troponin assays were done in seven patients and were positive in five patients indicating that myocarditis is a major contributor to shock, which is one of the commonest complications in KFD. Serum creatinine phosphokinase ranged from 656 to 23,000 U/L.

Conclusions

Altered sensorium was the most common alarming symptom that warrants referral to a higher centre. The major organ involvements that dominated the clinical presentation and course of illness were neurological complications, hypotension, significantly contributed by myocarditis and acute respiratory distress syndrome/pneumonitis. Encephalitis, myocarditis, ARDS and HLH were the major complications that caused mortality in our series. The elevated serum ferritin and the mortality associated with HLH described need further research to investigate the role of the macrophage system in the pathogenesis of severe KFD.

## Introduction

Kyasanur forest disease (KFD) or monkey fever is caused by Kyasanur forest disease virus (KFDV), a tick-borne virus. The spread of KFD in the states that share the Western Ghats like Karnataka, Kerala, Maharashtra, Goa and Tamil Nadu since 2012 has been well documented [1]. KFD has become an important differential diagnosis of acute febrile illness in patients living in the Western Ghats of South India [2]. KFD virus belongs to the *Flavivirus *genus (flavus meaning yellow) which consists of more than 70 viruses, most of which are arthropod-borne viruses. The other members in this genus are the viruses causing yellow fever, dengue, Japanese encephalitis, West Nile encephalitis, tick-borne encephalitis, and Zika fever [3]. The tick-borne encephalitis serocomplex includes some viruses that cause encephalitis syndromes like the Russian spring-summer virus and other viruses which cause haemorrhagic fever syndromes like Omsk haemorrhagic fever virus, Alkhurma haemorrhagic fever virus, and KFDV.

The first case of KFD in Kerala was detected in Noolpuzha, in the district of Wayanad, in the year 2013. This was preceded by the detection of KFD in Chamarajanagar district of Karnataka among forest workers in Bandipur Tiger Reserve [4] and among monkeys in Nilgiris district of Tamil Nadu [4] in 2012. Cases were detected in Wayanad district, which falls on the western side of the Western Ghats, and Malappuram district [5] which falls on the eastern side, in Kerala, in the year 2014. In 2015, a major epidemic erupted in Wayanad district, with 184 cases and nine deaths [6,7]. Anti-KFD immunoglobulin G antibody screening showed 33.9% positivity in Palakkad district of Kerala [8]. The initial case reports during and after the epidemic of 2015 were in the northeastern part of Wayanad, bordering Chamrajnagar in Karnataka and the Nilgiris in Tamil Nadu, but the recent cases are being reported from the northwestern part, bordering the Kannur district of Kerala. The non-reporting of human cases of KFD in the Nilgiris district of Tamil Nadu and the Palakkad district of Kerala needs further study and awareness programs, to find out whether the disease is being missed, as the Western Ghats are contiguous in all these geographical areas. The discontinuation of the formalin-inactivated KFDV vaccine in October 2022, though due to reasons of its ineffectiveness [9], can contribute to the establishment and the onward march of KFD in the state of Kerala [10].

This study seeks to familiarize physicians with the clinical presentation and the clinical, laboratory and imaging findings of the various complications of KFD. It also seeks to review the literature on the complications of KFD described, which will contribute towards the creation of a pathogenesis model of the disease.

## Materials and methods

This was a record-based retrospective study of the patients with KFD admitted to the Government Medical College Kozhikode from January 2013 to December 2023. Approval for the study from the institutional ethics committee was obtained before commencing the study. The diagnosis in these patients was made by the real-time reverse transcription polymerase chain reaction (rRT-PCR) assay targeting the KFDV NS5 gene in blood [11]. A total of 20 case records of patients with a diagnosis of Kyasanur forest disease between the periods of 2013 and 2023 were identified. The inclusion criteria for enrolling in the study was a positive KFDV real-time RT-PCR test result. Exclusion criteria used were as follows: patients who were diagnosed with KFD on clinical grounds and those with incomplete record data were excluded from the study. The final number of case records that were enrolled in the study was 12. Study parameters included sociodemographic details, clinical symptoms and signs, hematological, biochemical and radiological parameters and treatment details. As the district of Kozhikode has not reported any cases of KFD, all these patients were referred for tertiary care management from the neighbouring district of Wayanad. A case record form was used to enter the demographic details of the patient, symptoms, vital signs, and physical and systemic examination findings. The haematological and biochemical parameters were entered along with special investigation results whenever they were available. The imaging findings from chest X-ray, echocardiography, ultrasound examination, computed tomography (CT), magnetic resonance imaging (MRI) of the brain, and electroencephalogram (EEG) were also entered whenever these investigations were done. The treatment received by the patient was also recorded, along with the number of days of stay in the hospital, including the number of days on mechanical ventilation. For statistical analysis data was entered into an Excel sheet and frequency measurements of study variables were done using proportions and percentages.

## Results

Among the 12 patients whose records were available, seven (58.3%) were male and five (41.7%) were female, as described in Table 1. The first case in the study was in 2013, and the last case was in 2021. No further cases of KFD were reported till the end of 2023. All patients were of tribal ethnicity. Sickle cell anaemia in one patient and alcoholism in five patients were the comorbidities recorded. All patients had fever and headache. Loose stools were noted in eight patients. Oral ulcers were seen in four patients. Organomegaly (hepatomegaly, splenomegaly or lymphadenopathy) was seen in six patients. Auscultatory findings in respiratory system examination were seen in four patients. The total leukocyte count (TLC) ranged from 900 to 2400 c/mm3 except in one patient who had 7200c/mm3 (TLC: 4000-11,000 cells/mm3). Thrombocytopenia was seen in 10 patients. An elevated erythrocyte sedimentation rate (ESR) of more than 50 mm was seen in four out of nine patients (ESR <30 mm). Serum glutamic oxaloacetic transaminase (SGOT) ranged from 53 to 4860 U/L and serum glutamic pyruvic transaminase (SGPT) ranged from 41 to 1610 U/L SGOT:540 U/L; SGPT 5-40 U/L). Serum creatinine phosphokinase (CPK) was done in five patients and ranged from 656 to 23,000 U/L (CPK:10-120 mcg/L). Troponin I assay was done in seven patients and was positive in five patients (troponin I <0.03 ng/ml). Most patients were treated with intravenous fluids and symptomatic measures. Antibiotics, inotropes, anti-seizure medications, haemodialysis, non-invasive ventilation (NIV) and mechanical ventilation were used in appropriate patients. Case fatality rate was 42% (n=5) out of the 12 patients as shown in Table 2, of which, one patient was brought dead to the hospital, for whom an autopsy was performed, three patients expired in the hospital and one patient expired after she was referred back to a local hospital for step down care.

**Table 1:** Demographics, clinical, laboratory and imaging features and complications of 12 adults with KFD who were referred to Government Medical College Kozhikode, Kerala. BP= Blood pressure; RR= Respiratory rate; PR= Pulse rate; Hb=Haemoglobin, TLC= Total leukocyte count, aPTT= Activated partial thromboplastin time; ESR= Erythrocyte sedimentation rate; SGOT= serum glutamic oxaloacetic transaminase; SGPT=serum glutamic pyruvic transaminase; CPK= Creatinine phosphokinase; CSF= Cerebrospinal fluid; CRP= C Reactive protein; CXR= Chest X-Ray;  ECG= Electrocardiography; USG= Ultrasonography; ARDS= Acute respiratory distress syndrome; HLH= Hemophagocytic lymphohistiocytosis; AKI= Acute kidney injury; MRI= Magnetic resonance imaging; CT= Computed tomography; EEG= Electroencephalography. Normal ranges for laboratory studies: Hb 14-17 g/dL (men); 12-15 g/dL (women);  TLC 4000-11,000 cells/mm3; platelet count 1,50,000-4,00,000 c/mm3; ESR <30 mm/hr; aPTT 30-40 seconds;  INR less than 1.1;  Bilirubin 0.1-1.2 mg/dL;  SGOT 5–40 U/L; SGPT 5-40 U/L; Alkaline phosphatase 45-147 IU/L; Albumin 3.5-5.5 g/dL; Creatinine 0.7-1.3 mg/dL (men); 0.6-1.1 mg/dL (women); Potassium 3.5-5 mEq/mL; CPK 10-120 mcg/L;  CRP 0–10 mg/L; D-dimer <500 ng/mL; Triglycerides <150 mg/dL; Ferritin 12–300 ng/mL (men), 12–150 ng/mL (women);  Troponin I <0.03 ng/mL; Lipase 10-140U/L; CSF total count < 5 cells/mm3; CSF protein 15-40 mg/dL; CSF sugar 50-80 mg/dL

No	Age/ sex/ year	Symptoms	Co- morbidities	Clinical signs	Laboratory studies	Imaging studies	Complications	Treatment, days of hospital stay, outcome
1.	27/M 2019	Fever, headache, body ache, fatigue, loose stools, vomiting, altered sensorium.	Smoker alcohol intake	Blood presssure(BP): 120/80 mm of Hg,Respiratory rate(RR): 48/ mt, clubbing, bilateral basal crepitations,rhonchi, myoclonic jerks, neck stiffness, splenomegaly,	Hemoglobin(Hb) 9.5 g/dl Total leukocyte count (TLC) 900 c/mm3 platelet count 20,000 c/mm3, activated partial thromboplastin time (aPTT) 40 seconds, urine albumin 1+ Erythrocyte sedimentation rate (ESR) 25 mm, bilirubin 1.5 Serum glutamic oxaloacetic transaminase (SGOT) 53 U/L Serum glutamic pyruvic transaminase (SGPT) 45 U/L, cerebrospinal fluid (CSF) analysis - TLC 5 cells, lymphocytes 98%, protein 135 mg/dl, sugar 55 mg/dl, troponin 0.4 ng/ml, ferritin > 1000 ng/ml	Chest X Ray (CXR) Bilateral upper lobe infiltrate	Pneumonia, severe acute respiratory distress syndrome (ARDS), encephalitis, myocarditis, and hemophagocytic lymphohistiocytosis (HLH)	Mechanical ventilation for 5 days. Expired on day 10 of hospital stay.
2.	38/F 2020	Fever, headache, loose stools, vomiting, altered sensorium, hematuria.	Anemia	Pallor, hepatosplenomegaly, tachypnea, bilateral rhonchi, crepitations, bronchial breathing, generalized hypertonia.	Hb 7.6 g/dl, TLC 2,400 c/ mm3, plateletcount 47,000 c/mm3, aPTT 58 sec,ESR 66 mm. SGOT 489 U/L, SGPT 272 U/L, creatinine phosphokinase (CPK) 5442 U/L, troponin 16.3 ng/ ml, ferritin 1472 ng/ml, triglycerides 256, CSF analysis normal, creatinine 8 mg/dl.	CXR : Bilateral Infiltrates, Electrocardiography (ECG) :T inversion leads 1, aVL Moderate to severe Left ventricular dysfunction, Moderate ascites	ARDS, pneumonia, persistent shock, myocarditis, acute kidney injury ( AKI), HLH, rhabdomyolysis, ventilator associated pneumonia	Mechanical ventilation for 10 days Hospital stay : 25 days. Expired after 5 days in the peripheral hospital.
3	44/M 2015	Fever, headache, body ache, fatigue, altered sensorium, seizures.	Alcohol intake, smoking	BP: 90/60 mm of Hg, Oxygen Saturation(SpO2): 60%, oral ulcers, neck stiffness.	Hb 13.9 g/dl, TLC 2200 c/mm3, platelet count 60,000 c/mm3, urine routine : sediments +, albumin +, ESR 5 mm, bilirubin 1.1 mg/dl, SGOT 330 U/L, SGPT 531 U/L, creatinine 3.2 mg/dl, CSF: dry tap.	ECG: normal	Encephalitis, resistant shock, myocarditis, AKI, hepatitis hyponatremia	Expired
4	44/F 2015	Fever, headache, body ache, fatigue, sore throat, vomiting, cough, altered sensorium,seizures, bleeding.	Nil	Pulse rate(PR): 68/ mt, BP: 90/60 mm of Hg.	Hb 8.4 g/dl, TLC 1000 c/mm3, platelet count 35000 c/mm3, ESR 15 mm, bilirubin 0.7 mg/dl, SGOT 610 U/L, SGPT 815 U/L, creatinine 0.8 mg/dl, CSF: dry tap	ECG: tachycardia	Encephalitis, persistent shock, myocarditis, and hepatitis	Expired
5	28/M 2020	Fever, headache, prostration, bodyache, loose stools, vomiting, altered sensorium, abdominal pain.	Alcohol dependence syndrome	Tremors, blepharitis, subconjunctival hemorrhage, conjunctival congestion, BP: 90/60 mm of Hg, hepatomegaly	Hb 12.8 g/dl, TLC 1900 c/mm3, Platelet count 2,03,000 c/mm3, creatinine 3.3 mg/dl, urine albumin 3+ S.CPK 19,000 U/L, SGOT 813 U/L, SGPT 341 U/L. CSF Normal.		Shock, rhabdomyolysis, AKI.	Survived. Hospital stay for 7 days.
6.	20/F 2016	Fever, bodyache, prostration, altered sensorium.	Nil	Oral ulcers, facial puffiness. BP 90/70 mm of Hg, chest bilateral rhonchi, crepitations	Hb 11.6 g/dl, TLC 2000 c/mm3, platelet count 1,10,000 c/mm3, ESR 4 mm, bilirubin 3.9 mg/dl, SGOT 650 U/L, SGPT 810 U/L, Alkaline phosphatase 652 U/L, albumin 2.2 g/dl, creatinine 0.7 mg/dl, CSF: normal.	Computed tomography (CT) head : normal, CXR: bilateral interstitial pattern.	Encephalitis/ encephalopathy, pneumonia, hepatitis.	Survived Hospital stay for 10 days.
7.	22/F 2016	Fever, headache, bodyache, fatigue,loose stools, vomiting, altered sensorium, 2 episodes of generaalised tonic clonic seizures, abdominal pain, gum bleeding.	Nil	Oral ulcers, lymphadenopathy, BP: 80/60 mm of Hg, hepatomegaly, right iliac fossa tenderness.	Hb 11.6 g/dl, TLC 1600 c/mm3, platelet count 15000 c/mm3, International normalised ratio of prothrombin time (INR) 2.1seconds, ESR 124 mm of Hg, bilirubin 2.1 mg/dl, SGOT 4860 /L, SGPT 1610 U/L, Creatinine 0.6 mg/dl, potassium 3.0 meq/dl, troponin 0.08 ng/ml, lipase 753 U/L, CPK 23,000 U/L.	Ultrasonogram (USG) abdomen: few subcentimeter lymph nodes with hyperechoic mysentry, hepatomegaly 16 cm.	Encephalitis, myocarditis, and persistent shock pancreatitis peritonitis, rhabdomyolysis, hyperglycemia, hypokalemia.hepatitis.	Survived. Mechanical ventilation for 3 days,
8.	29/M 2021	Fever headache, bodyache, fatigue, loose stools, vomiting.	Sickle cell anemia, COVID 19 positive.	Icterus, BP: 100/70 mm of Hg, SpO2 89 %	Hb 8.5g/dl, TLC 1200 c/mm3, platelet count 2,13,000 c/mm3, aPTT 60 seconds, bilirubin 3.1 mg/dl, SGOT 296 U/L, SGPT 89 /L, Creatinine 0.7 mg/dl, CSF: normal study, ferritin 1000 ng/ml, CPK 656 U/L.	ECG : normal CT brain: normal	Hepatitis	Survived Hospital stay for 10 days.
9.	43/M 2021	Fever, headache, body ache, fatigue, loose stools vomiting, cough, altered sensorium.	History of snake bite, smoker alcohol intake	PR: 55/ mt, BP: 80/60 mm of Hg, RR: 30 / mt, conjunctival congestion, clubbing, thigh muscle tenderness, bilateral crepitations, hepatomegaly.	Hb 13.3 g/dl, TLC 7200 c/mm3, platelet count 77,000 /mm3, aPTT no coagulation , ESR 20 mm , bilirubin 1.4 mg/dl, SGOT 281 U/L, SGPT 81 U/L, creatinine 5.8 mg/dl, triglycerides 175 mg/dl, troponin 0.15 ng/ml, CPK 886 U/L, C reactive protein (CRP) 16 mg/L, glycated hemoglobin (HbA1c) 6.3 %, Artereal blood gas analysis Metabolic acidosis, RBS 392 mg/dl, peripheral smear : giant platelets.	ECG: ST elevation 2, 3, avf , v2- v5, CXR bilateral ill defined opacities , hyperinflated lung fields, Echocardiogram: global left ventricular hypokinesia.	Moderate ARDS, pneumonia, persistent shock, myocarditis AKI lower motor neuron weakness and numbness of bilateral upper limb, stress hyperglycemia	Survived. Hospital stay for 10 days.
10.	19/M 2019	Fever, headache, body ache, fatigue, loose stools, cough, hemoptysis, knee joint pain, breathlessness.	Nil	Conjunctival congestion, calf muscle tenderness, clubbing, BP: 80/60 mm of Hg, RR: 56 / Mt, bilateral crepitations.	Hb 6.3 g/dl, TLC 2300 c/mm3, platelet count 3.1L c/mm3, aPTT 38 seconds, ESR 115 mm , bilirubin 4.1 mg/dl, SGOT 55 U/L, SGPT 41 U/L, albumin 3.0 g/dl, S. creatinine 1.6 mg/dl, ferritin 620 ng/ml.	ECG normal. CXR bilateral patchy infiltrates, CT thorax ARDS diffuse patchy consolidation, ground glass opacities in dependent hilar and peripheral regions, Echocardiogram: good left ventricular function.	Moderate ARDS, diffuse alveolar hemorrhage, pneumonia, persistent shock.	Non invasive ventilation Survived
11.	18/M 2013	Fever, headache, sore throat, altered behavior, loose stools, vomiting,cough, hemoptysis, malena.	Monkey deaths in vicinity, forest visit.	Conjunctival congestion, oral ulcers,small cervical nodes, PR: 68/mt, BP:100/60 mm of Hg, hypotonia of all limbs, sluggish deep tendon reflexes, bilateral plantar extensor.	Hb 14.3 g/dl, TLC 1700 c/mm3, platelet count 40,000c/mm3, ESR 80 mm, SGOT 1313 U/L, SGPT 824 U/L, bilirubin 2 mg/dl, CSF normal.	Electroencephalogram (EEG) : diffuse cerebral dysfunction, Magnetic resonance imaging (MRI)brain : altered signal intensities in temporoparietal region.	Encephalitis, hepatitis,	Survived. Became febrile again in the second week of admission. Biphasic illness
12	45/M 2/2015	Fever for 4 days. Brought dead to hospital.	Alcohol intake	Conjunctiva congested, subconjunctival hemorrhage in right eye.	Hb 13g/dl, TLC 2000 c/mm3, neutrophils 69%, platelet count 82,000 c/mm3, bilirubin 1.5 mg/dl, SGOT 2330 U/L, SGPT 580 U/L.	Autopsy showed copious amount of blood stained frothy fluid in the airways. Both lungs were congested and edematous with subpleural hemorrhage in the undersurface. Liver and spleen surface showed scattered petechial hemorrhages. Kidneys pale with loss of corticomedullary differentiation and cortical streaks of hemorrhage. Brain was congested. Histopathological examination showed pulmonary edema and patchy foci of bronchopneumonia in the lung. Liver showed mild steatosis and portal inflammation. Kidneys and spleen were congested.		

**Table 2:** Complications observed in patients with Kyasanur Forest Disease The patient who was brought dead to the hospital was not included in the analysis of complications, but was included in the calculation of case fatality rate. Hepatitis was defined as S. Bilirubin >1.3 mg/dL or AST/ALT > 400 U/L. Acute kidney injury was defined as Creatinine >1.5 mg/dL. ARDS= Acute respiratory distress syndrome; HLH= Hemophagocytic lymphohistiocytosis.

No.	Complication	Frequency n=11(%)
1.	Altered sensorioum	9 ( 82%)
2.	Persistent shock	7 (64%)
2.	Encephalitis	6 (55%)
3.	ARDS	6 (55%)
4.	Myocarditis	6 (55%)
5.	Bleeding manifestations	6 (55%)
6.	Hepatitis	6(55%)
7.	Acute kidney injury	4 (36%)
8.	Rhabdomyolysis	3 (27%)
9.	HLH	2 (18%)
10.	Stress hyperglycemia	2 (18%)
11.	Pancreatitis	1 (9%)
12.	Peritonitis	1 (9%)
13.	Mortality	5(42%)

## Discussion

The study looked at the various complications seen in patients with KFD who were referred to a tertiary care hospital in north Kerala over a period of 11 years. The age group was between 18 and 44 years, indicating that young working people are more vulnerable. All the patients in this case series belonged to tribal ethnic groups. Most of the people affected by KFD in the previous studies [12,13,14] were either frequent forest visitors or people of tribal ethnicity. A study that assessed the risk factors for being infected with KFDV in the tribal population found that 74% of the people belonging to tribal ethnic groups do not use footwear, and more than 88% do not cover their body or use gloves or tick repellents while working in the forest [15]. This emphasizes the need for public health intervention to provide the basic protection gear to the people of tribal ethnicity who live in close proximity to the forest, and who are most vulnerable to KFD.

The study is notable for the diverse complications seen in patients with KFD. All patients had at least one organ involved. Most patients had multiple organ involvement. In a previous study by Gladson et al [14] in 2021 which was a community-based study conducted using data from primary and secondary hospitals, any organ involvement, including bleeding manifestations, was seen only in 34%. The major organ involvements that dominated the clinical presentation and course of illness were encephalitis, Acute respiratory distress syndrome (ARDS)/pneumonia and myocarditis.

Our series has a relatively high incidence of neurologic complications, especially encephalitis (55%). Altered sensorium was seen in nine patients, in whom evidence for meningoencephalitis (like neck stiffness, seizures unexplained by metabolic derangements, abnormalities in cerebrospinal fluid analysis (CSF) and EEG were seen in six patients. Three of these patients succumbed to the illness. Seizures, tremors, myoclonic jerks, hypertonia, hypotonia, decreased deep tendon reflexes, and bilateral extensor plantar response were some of the other neurological examination findings recorded. Seizures occurred in three patients. CSF study was done in six patients and revealed normal values except in one patient who had 5 cells, 98% lymphocytes, and 135 mg/dl of protein. Adhikari Prabha et al also reported normal CSF in seven out of their 10 cases. Only mild lymphocytosis and protein elevation were seen in the other cases. They also reported no evidence of encephalitis but cerebral oedema in two out of five autopsies [12]. The autopsy performed on one patient reported a congested brain. EEG was done in one patient who had developed seizures, which showed diffuse cerebral dysfunction. MRI done in the same patient showed altered signal intensities in the temporoparietal regions. Adhikari Prabha et al reported altered sensorium in 45% and seizures in 24% [12]. Gupta et al [16] in a study of 297 patients, reported altered sensorium and seizures as the clinical symptoms and focal infarcts as the major radiological findings in the first phase. In the second phase, the common symptoms were cerebellar signs and vision abnormalities. Leptomeningeal enhancement was the most common radiological abnormality.

Haemorrhagic manifestations were recorded in six patients (55%). Haematuria, haemoptysis, melena, gum bleeding, and subconjunctival haemorrhages were observed. Bleeding manifestations recorded in the studies by Gupta et al [13] and Gladson et al [14] in 2021, were 8% and 20%, respectively, as shown in Table 3. Bleeding manifestations are attributed to the activation of the vascular endothelium. Hemophagocytic lymphohistiocytosis (HLH) developed in two patients, as per the modified HLH 2009 criteria. Both these patients succumbed to the illness. This finding was unique in our series, as this has not been described in KFD. Serum ferritin was elevated (above 500 ng/ml) in four patients in whom it was tested and was above 1000 ng/ml in three of them(ferritin 12-300 ng/ml). These findings point towards hyperinflammation, probably mediated by a cytokine storm. Iyer et al [17, 18] in 1959 in an autopsy study, described moderate to marked prominence of reticuloendothelial elements in the liver and spleen with pronounced erythrophagocytosis. Webb et al [19] described phagocytosis of RBCs and nuclear material in the peripheral blood in their necropsy study in Macaca radiata.

**Table 3:** The proportion of complications in the major descriptive studies on Kyasanur Forest Disease Adhikari Prabha et al. [12]; Gupta et al. [13]; Gladson et al [14] ARDS= Acute respiratory distress syndrome; HLH= Hemophagocytic lymphohistiocytosis Acute kidney injury was defined as serum creatinine > 1.5 mg/dL

No,	Study	Year	Setting	Complications	Number	Percentage
1.	Adhikari Prabha et al	1992	n=100	Hypotension	61	61%
				Respiratory signs of bronchopneumonia	45	45%
				Altered sensorium	45	45%
				Bleeding manifestation	54	54%
				Mortality	33	33%
2.	Gupta et al	2021	n=192	Hypotension	25	13%
				Infiltrates on Chest X-Ray	22	11%
				Altered sensorium	25	13%
				Bleeding manifestations	16	8%
				Bloodstream infection	5	2%
				Myocarditis	11	6%
				Pancreatitis	8	4%
				Mortality	17	9%
3.	Gladson et al	2021	n=60	Neurological complications	14	24%
				Altered sensorium	9	15%
				Hemorrhagic manifestations	12	20%
				Persistent shock	12	20%
				Acute kidney injury	6	10%
				Pneumonitis	2	3%
				Mortality	4	7%

The primary cardiovascular manifestation in the study was persistent hypotension seen in seven patients. Hypotension in KFD can be due to increased vascular permeability and myocarditis [20], although dehydration also contributes. Myocarditis was diagnosed in six of these patients, based on troponin positivity, and electrocardiographic or echocardiographic abnormalities. Echocardiogram showed severe left ventricular dysfunction in one patient and global left ventricular hypokinesia in another. Four patients with myocarditis succumbed to the illness. In the study by Gupta et al [13], hypotension at presentation and myocarditis were associated with an increased risk of mortality. Adhikari Prabha et al reported that out of four patients in whom the myocardium was examined in autopsy, two showed evidence of myocarditis (interstitial infiltration of round cells) [12].

The respiratory system complications observed were pneumonia/ARDS in five patients. All patients had bilateral lung infiltrates in the chest radiograph. High-resolution computed tomography scan of the thorax was done in one patient, which showed diffuse patchy areas of consolidation and ground glass opacities in the dependent, hilar, and peripheral regions suggestive of ARDS. 3 patients were mechanically ventilated, of whom two succumbed to the illness. Autopsy done on one patient showed blood-stained frothy fluid in the airways along with congested and oedematous lung and subpleural haemorrhage. Iyer et al also described blood-stained pleural effusion and haemorrhagic pneumonia in both gross and microscopic examinations in their autopsy studies [17,18]. Haemorrhagic pulmonary oedema in two out of three post-mortem cases was reported by Adhikari et al [12]. Respiratory involvement was reported in 3.3% to 45% in previous studies [12-14].

Elevated transaminases above 400 U/L were recorded in six patients, of which two had values above 1000 U/L. Two of them had features of acute liver failure. SGOT more than SGPT observed in five patients is characteristic and described elsewhere [13]; the reason being the contribution of SGOT from cardiac muscles due to myocarditis and skeletal muscles due to rhabdomyolysis [20]. In our series, rhabdomyolysis was observed in four patients, manifested as extremely high CPK values. In the study by Gupta et al [13], elevated CPK was significantly associated with mortality.

Acute kidney injury (AKI) was observed in four patients among which two succumbed to the illness. This was also a unique observation in our series. AKI is attributed to hypovolemia and rhabdomyolysis. Two patients with AKI also had rhabdomyolysis. One patient underwent five sessions of haemodialysis. In the study by Gladson et al [14], the prevalence of AKI in KFD was 10%, where serum creatinine was significantly higher in nonsurvivors compared to survivors.

Two studies have looked at the predictors of mortality in KFD. Higher age at presentation, altered sensorium, myocarditis, hypotension, low platelet count, elevated transaminases, activated partial thromboplastin time and CPK were associated with mortality in the study by Gupta et al [13]. Advanced age, altered sensorium, seizures, and the presence of comorbidities were associated with mortality in the study by Gladson et al [14]. Encephalitis, myocarditis, ARDS and HLH were the major complications that caused mortality in our series. Bleeding manifestations and haemorrhages were not reported to be associated with mortality in both studies.

The study has limitations associated with its retrospective design. The small sample size may not have captured the full range of complications. The reason for the greater incidence of complications in the study is because of the referral bias, as all the patients were referred for tertiary care. Hence the study describes the profile of severe KFD and is different from what will be seen in a primary or secondary care setting. 

## Conclusions

This study seeks to familiarize clinicians with the clinical, laboratory and imaging findings of the various complications of KFD. Altered sensorium was the most common alarming symptom described that may warrant referral to a higher centre. The major organ involvements that dominated the clinical presentation and course of illness were neurological complications and hypotension - significantly contributed by myocarditis and ARDS/pneumonitis. Encephalitis, myocarditis, ARDS, and HLH were the major complications that caused mortality in our series. The elevated serum ferritin and the mortality associated with HLH described need further research to investigate the role of the macrophage system in the pathogenesis of severe KFD.
